# Assessing Physical Activity and its Relationship to Cardiovascular Risk Factors: NHANES 2003-2006

**DOI:** 10.1186/1471-2458-11-387

**Published:** 2011-05-25

**Authors:** Amy Luke, Lara R Dugas, Ramon A Durazo-Arvizu, Guichan Cao, Richard S Cooper

**Affiliations:** 1 Department of Preventive Medicine and Epidemiology, Loyola University Stritch School of Medicine, 2160 S. 1st Ave Maywood, IL, 60153, USA

## Abstract

**Background:**

Levels of physical activity (PA) in the general population are difficult to characterize. Historically measurement has been based on self-report, which can be subject to bias. PA monitor use has created opportunities to improve surveillance and analytic research on activity and health. The aims of the current study were to investigate the associations between objectively measured PA and cardiovascular disease risk factors and obesity.

**Methods:**

Data on PA from accelerometers, demographics, blood pressure, plasma glucose and lipids, self-reported hypertension and diabetes were obtained for adults, ages 20-65, in the NHANES surveys, 2003-2006. Outcomes were assessed as levels of moderate and vigorous activity, percentage of participants meeting recommended guidelines, and the correlations between activity and cardiovascular risk factors. Accelerometry data were available on 3,370 adults. Based on standard algorithms, activity levels were extremely low in all age-gender-race/ethnic groups, with an average of only 1 bout of vigorous activity lasting longer than 1 minute/day.

**Results:**

Men spent 35 minutes in moderate activity/day, women 21 minutes; >75% of this activity was accumulated in 1-minute bouts. Levels of activity declined sharply after age 50 in all groups. Negative associations were observed between minutes of combined moderate and vigorous activity and systolic blood pressure, blood glucose, diabetes, hypertension, body mass index and obesity, and a positive association was seen with HDL-cholesterol (all P ≤ 0.03), suggesting valid rank ordering of participants by activity level.

**Conclusion:**

The magnitude of the gap between self-report and accelerometry activity must be a result of either a vast social acceptability bias in reporting or inaccurate measurement with accelerometry. Therefore, due to the low validity of self reported PA data for epidemiologic research, it is pertinent to encourage the use of valid, objective methods to assess PA.

## Background

The population burden attributable to cardiovascular disease (CVD) has evolved rapidly over the last 50 years. Vigorous efforts to improve levels of smoking, hypercholesterolemia and uncontrolled hypertension have contributed significantly to the 75% reduction in CVD mortality in the US since 1968 [1,2]. This improvement in lifestyle continues and several goals for *Healthy People 2010 *for heart disease and stroke risk factors have already been met [3]. As is well recognized, however, significant adverse trends in obesity and diabetes have emerged and threaten to erode these gains [4]. The new urgency attached to the prevention of obesity and its sequelae has furthermore heightened interest in potential strategies that can limit age-related weight gain [5]. Unfortunately, despite the relatively straightforward nature of the energy equation, balancing calorie intake with expenditure has become difficult for large segments of the population in the current obesigenic environment. In an attempt to address the obesity epidemic, considerable emphasis has therefore been placed on surveillance of physical activity and the initiation of campaigns to enhance leisure activity [6,7]. These strategies depend on accurate information about patterns and levels of energy expenditure in the general population.

In contrast to the "classic" CVD risk factors, until recently assessment of physical activity in large population studies has relied on self-reported questionnaires while direct measurement has been feasible only in relatively small validation studies [8,9]. Self-report of behaviors such as alcohol intake, diet and physical activity are notoriously unreliable, being subject to both random and systematic reporting bias [10-12]. Development of improved physical activity monitors, i.e., accelerometers, has now made it possible to measure activity patterns objectively in free-living individuals [13-15]. Doubly labeled water (DLW), a non-invasive method of indirect calorimetry [16], has also been applied on a limited scale to field research [17,18], however, it remains prohibitively expensive for most large studies, and accelerometers are likely to be the most widespread technique that will be used for the foreseeable future.

In the 2003-2006 NHANES surveys participants wore an accelerometer for 7 consecutive days. In this analysis we examine the patterns of physical activity in the 3,370 individuals between 20 and 65 years with adequate quality data and non-debilitating illnesses and assess the relationship between activity and CVD risk factors and obesity.

## Methods

Data from NHANES 2003-2006 were used in these analyses. Using a complex, multistage probability sampling design, NHANES recruited a representative sample of the total civilian non-institutionalized population, 2 months and older, in the United States. Briefly, NHANES participants perform an at-home interview and, approximately two weeks later, a clinic examination at a mobile examination center (MEC). The examination lasts approximately 3-4 hours and site-specific error is minimized by standardizing all data-collection methods [19]. All participants provide informed consent for both the at-home interview and MEC examination and the US Department of Health and Human Service approved the study. During NHANES 2003-2006, a total of 20,470 individuals were interviewed and examined (all ages). Of 7,501 individuals between the ages of 20 and 65, 4,463 individuals wore the activity monitor for a minimum of 4 valid wear days. There were 1,093 adults excluded for one of the following reasons: missing BMI data (n = 31), pregnant at time of examination (n = 240), self-reported race/ethnicity other than non-Hispanic white, non-Hispanic black or Mexican American (n = 343), or self-reported chronic health condition that might have impacted physical activity levels, such as congestive heart failure, stroke or emphysema (n = 542). Thus, the analytical sample included 3,370 adults between 20 and 65 years of age.

Weight (kg) and height (m) were measured according to standard procedures and used to calculate body mass index (BMI, kg/m^2^). Brachial SBP and DBP measurements were made during a single examination and following standard procedures; the average value for up to three measurements was used [20].

Blood was drawn following an overnight fast and processed according to standard procedures [21]; plasma glucose and lipids concentrations were measured. Plasma glucose was assessed and triglycerides and HDL-cholesterol were measured in serum (Roche Diagnostics, Indianapolis, IN), while levels of LDL-cholesterol were calculated using the Friedewald formula [21].

Physical activity monitors (Actigraph model 7164; Actigraph LLC; Fort Walton Beach, FL) were placed on all ambulatory participants six years and older [22]. The Actigraph records vertical accelerations as "counts" representing the relative intensity of each movement [23]. The monitor was worn over the right hip; participants were asked to wear the monitor while awake and remove it for swimming or bathing [15]. Data were summarized in 1-minute epochs for 7 days.

For the purpose of our analyses, we included accelerometer data from 3,370 healthy individuals, 20-65 years of age, who met our minimum wear time criteria, as described below. Each participant was required to achieve a minimum of 10 hrs of monitor wear on four or more days. Wear time was determined by subtracting non-wear time from 24 hr, while non-wear time was defined as an interval of ≥60 consecutive minutes with zero activity counts allowing for intervals of 1-2 minutes of relatively low activity counts, i.e., 1-100 counts [15]. The average number of valid wear days was 5.97 for men and 5.94 for women.

We defined moderate and vigorous physical activity cut-points for adults using previously published recommendations [24], as described by Troiano [15]. Briefly, cut-points allow the conversion of accelerometer counts into estimates of activity intensity, i.e., moderate or vigorous, using weighted averages. The cut-point was 2020 counts/min for moderate activity and 5999 counts/min for vigorous; these were used to estimate the total number of minutes per day spent in each activity level. We present physical activity data as the total time in minutes accumulated in either 1- or 10-minute intervals of moderate, vigorous or moderate plus vigorous activity, as well as activity counts per minute (ct/min). Following prior conventions, we allowed for up to 2 minutes of below threshold count activity before considering the bout to be ended [15]. For these analyses, therefore, the continuous measure of "counts over time" was converted into "bouts" and these became the unit of analysis.

As a form of sensitivity analysis, we repeated the estimates of time spent in moderate or vigorous activity using cut-points which progressively reduced the counts per minute down to a minimum of 30% of the pre-set standards described above [15].

Participants were identified as diabetic if they self-reported having the condition or were taking insulin or oral hypoglycemic medications. Participants were identified as hypertensive if they were taking anti-hypertensive medication or their systolic blood pressure (SBP) was ≥140 or diastolic blood pressure (DBP) was ≥90. We used standardized BMI cut-points to categorize participants as either overweight (BMI ≥25 and <30 kg/m^2^) or obese (BMI ≥30 kg/m^2^)[22].

### Statistical Methods

Data are presented as the total time accumulated in either 1- or 10-minute bouts of activity level, mean counts per minute and mean wear time. Weighted means and standard errors are computed for each of the continuous variables of interest, and proportions with standard errors are reported for the categorical variables by sex and race/ethnicity, accounting for the complex sampling scheme used by NHANES. Plasma glucose concentrations were adjusted using the fasting sampling weights included in the NHANES dataset. Partial correlation coefficients were computed for each of the physical activity measures with well-established CVD risk factors, BMI, overweight and obesity accounting for age, gender and race/ethnicity. Standardized coefficients were used to express change in the dependent variable per standard deviation in the independent variables. Summary statistics and correlation coefficients were calculated using SUDAAN (v.9, Research Triangle Park, NC), and Stata SE (v.11, College Station, TX).

## Results

The characteristics of the 3,370 participants' ages 20-65 years are presented in Table 1. The mean age of Mexican-American participants was somewhat lower than for the other racial/ethnic groups (i.e., 36 years vs. 42 for whites and 39 for blacks). As anticipated, overweight and obesity were common in all groups, with a substantially higher prevalence in blacks. In addition, blacks had higher blood pressures and lower total cholesterol and LDL-C levels. Diabetes prevalence, unadjusted for age, was higher in blacks (8.0%) than either whites (3.6%) or Mexican Americans (5.0%), as was hypertension prevalence (42.9% vs. 22.4% and 12.5%, respectively).

**Table 1:** Characteristics of Participants by Gender-Race/Ethnic Groups (mean, SE) - NHANES 2003-2006, ages 20-65 y (n = 3,370)

Variable	White (N = 1763)		Black (N = 761)		Mexican American (N = 846)		Total (N = 3370)	
	Mean	SE	Mean	SE	Mean	SE	Mean	SE
Male (%)	51.8	1.1	47.5	2.0	56.9	1.5	51.7	1.0
Age (y)	41.7	0.4	39.0	0.4	36.2	0.6	40.8	0.4
Height (cm)	171.6	0.2	169.8	0.4	164.9	0.3	170.7	0.2
Weight (kg)	82.3	0.6	85.9	1.2	77.1	0.8	82.2	0.5
Body Mass Index	27.9	0.2	29.8	0.4	28.3	0.3	28.2	0.2
Overweight (%)	32.9	1.1	33.5	2.1	40.1	1.7	33.7	1.1
Obese (%)	29.9	1.5	42.9	2.4	31.4	1.9	31.7	1.3
Hypertensive (%)	22.4	1.3	30.0	2.1	12.5	1.7	22.4	1.1
Diabetic (%)	3.6	0.5	8.0	1.1	5.0	0.8	4.3	0.5
SBP (mmHg)	119.5	0.5	123.2	0.8	117.7	0.6	119.8	0.5
DBP (mmHg)	72.3	0.3	72.5	0.5	69.6	0.5	72.0	0.3
Total Cholesterol (mg/dL)	200.3	1.1	191.7	1.5	196.8	1.5	198.8	0.8
HDL-cholesterol (mg/dL)	54.5	0.4	56.7	1.0	50.1	0.5	54.3	0.3
LDL-cholesterol (mg/dL)	110.6	1.6	107.6	1.9	114.0	1.9	110.6	1.2
Triglyceride (mg/dL)	131.3	3.2	116.1	5.4	147.1	9.9	131.0	2.9
Plasma Glucose (mg/dL)*	99.1	1.0	102.1	2.0	104.0	1.9	99.9	0.9

*Fasting sampling weights were used for plasma glucose concentrations. Plasma glucose sample sizes: n = 771 white, n = 306 black, n = 370 Mexican American, n = 1447 total sample

**Figure 1:**
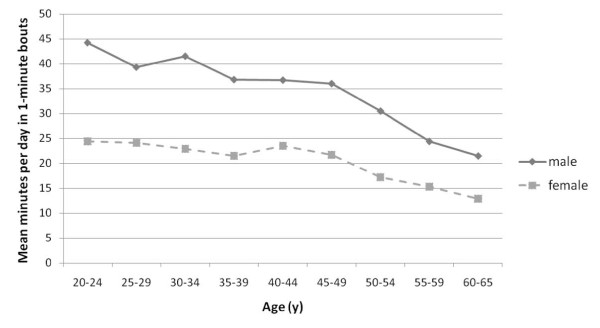
**Mean number of minutes per day of moderate plus vigorous activity combined in 1-minute bouts by 5-year age groups - NHANES 2003-2006 (n = 3,370)**.

Physical activity was summarized as the mean number of counts per minute (cts/min) and in the categories of moderate or vigorous, presented in 1-minute and 10-minute bouts (Table 2). The average counts were higher among men than women (393 vs. 321 ct/min), and highest among Mexican-American men and women. As anticipated, activity declined sharply after age 50 (Figure 1). Vigorous activity was recorded very infrequently; no gender-race groups had ≥1 bout of vigorous activity per day that lasted at least 10 minutes (Table 2). Even vigorous activity bouts of 1 minute were rare, ranging from 0.4/day among Mexican American women to 1.5/day among white men. Daily moderate activity was recorded in 32 to 41 bouts of 1-minute duration in men, and 18 to 20 1-minute bouts in women. Given the potential for artifact, the significance of 1-minute bouts of activity is difficult to interpret. The most stable estimate of activity patterns may therefore be the number of minutes per day spent in 10-minute bouts of activity. This measure ranged from a high of 10.3 minutes per day of moderate plus vigorous activity among Mexican-American men to a low of 5.0 minutes among Mexican-American women, with a population average of 6.6 minutes per day. Furthermore, over 66% of men and 68% of women did not accumulate a single 10-minute bout of moderate or vigorous activity on any of the days.

**Table 2:** Physical Activity Monitor Measures by Gender-Race/Ethnic Groups (mean, SE)* - NHANES 2003-2006, ages 20-65 y (n = 3,370)

Variable	Male								Female							
	
	White (N = 926)		Black (N = 386)		Mexican American (N = 463)		Total (N = 1775)		White (N = 837)		Black (375)		Mexican American (N = 383)		Total (N = 1595)	
	Mean	SE	Mean	SE	Mean	SE	Mean	SE	Mean	SE	Mean	SE	Mean	SE	Mean	SE
Mean Counts per min	383.6	5.0	374.4	8.7	466.4	9.7	392.7	4.3	324.7	4.1	297.9	6.6	328.1	5.6	321.0	3.5
Mean Wear Time (hr/d)	14.6	0.1	14.9	0.1	14.3	0.1	14.6	0.1	14.2	0.1	14.3	0.1	14.0	0.1	14.2	0.1
Mod Activity (min/d in 1-min bouts)	33.2	0.9	31.8	1.4	40.9	1.5	34.1	0.8	20.3	0.7	18.0	1.3	18.7	0.9	19.9	0.7
Vig Activity (min/d in 1-min bouts)	1.5	0.2	1.5	0.3	1.3	0.3	1.5	0.1	1.3	0.1	0.6	0.1	0.4	0.1	1.1	0.1
Mod & Vig (min/d in 1-min bouts)	34.7	0.9	33.4	1.4	42.1	1.5	35.6	0.8	21.6	0.8	18.5	1.3	19.1	0.9	21.0	0.7
Mod Activity (min/d in 10-min bouts)	7.0	0.5	7.5	0.8	8.9	0.6	7.4	0.4	5.3	0.5	4.7	0.8	4.5	0.4	5.2	0.4
Vig Activity (min/d in 10-min bouts)	0.8	0.2	0.7	0.3	0.6	0.2	0.8	0.1	1.0	0.1	0.4	0.1	0.1	0.04	0.8	0.1
Mod & Vig (min/d in 10-min bouts)	8.7	0.6	9.2	0.9	10.3	0.6	9.1	0.5	6.9	0.6	5.3	0.9	5.0	0.4	6.6	0.5

*Age-adjusted by year 2000 US population

Abbreviations: Mod, moderate; Vig, vigorous

**Figure 2:**
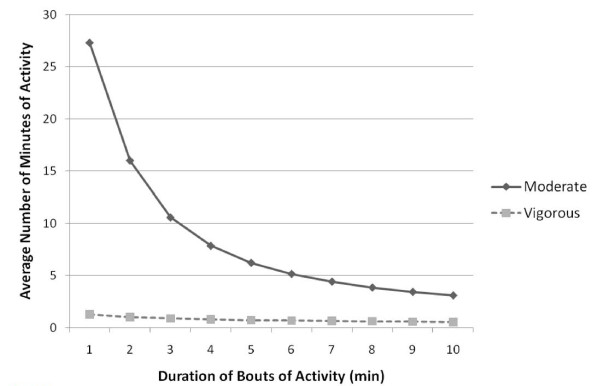
**Number of minutes accumulated in continuous bouts of moderate or vigorous activity lasting from 1 to 10 minutes, NHANES 2003-2006, (n = 3370)**. The number of daily bouts was determined by averaging all bouts of the specified duration that were accumulated over valid wear days (i.e., number of bouts accumulated on all valid wear days/number of valid wear days; average valid wear days = 5.95)

Previous national prevalence estimates of activity patterns have come exclusively from questionnaires on discrete behaviors and have suggested that relatively large proportions of the population meet current physical activity guidelines [8,25,26]. In contradiction, activity as measured by activity monitors demonstrated that guidelines are rarely being met. Thus, only 0.2% of women and 0.4% of men in the NHANES sample would have met current guidelines requiring a minimum of approximately two 10-minute blocks of moderate activity per day [6]. While more individuals accumulated greater amounts of total moderate activity, the overwhelming majority of this time was accounted for by 1-minute bouts (i.e., 75%); the time spent in longer bouts declined rapidly (Figure 2). Across the entire 6-day average period of recording, only 14% had a single block lasting 20 minutes.

Quantification of physical activity using accelerometry depends critically on the number of counts per minute used as the cut-point defining moderate or vigorous activity. Accelerometers can be well calibrated for walking or running but may miss other forms of body motion thereby underestimating intensity. As a sensitivity analysis we therefore reduced the number of counts required to qualify as moderate/vigorous activity in a stepwise fashion from 80% of the laboratory-validated standard down to 30%, and repeated the analyses above. Current guidelines of two 10-minute blocks of moderate activity per day were met by 1.9% of the population with the threshold reduced to 80% of the standard and 29.5% when the threshold was only 30% of the standard. Most importantly, the majority of time in activity (~ 90%) was still spent in 1 minute bouts, suggesting sustained fitness-inducing exercise continued to be rare even at this much reduced threshold of counts per minute.

As a means of evaluating the internal validity of the monitoring procedure we examined the relationship between activity measures and primary cardiovascular risk factors. In a combined analysis, with adjustment for age, gender and race/ethnicity, both activity counts per minute and number of minutes in 1-minute bouts were significantly negatively associated with SBP, blood sugar, hypertension and diabetes, while activity measures were positively associated with HDL-cholesterol (Tables 3 &4). Table 4 presents the change in risk factor based on a standard deviation change in the activity measure; as a proportion of the mean for the given risk factor, the largest effect was seen for HDL-C, followed by glucose. In Table 4, the association with hypertension and diabetes is further summarized as the change in odds ratio; for hypertension the odds were 20% lower, and for diabetes 35% lower, with one standard deviation increase in activity. After removing the effect of BMI and age, a negative association remained for plasma glucose in the total sample and the majority of sub-groups (Table 5). Negative associations were also seen for diabetes, hypertension and HDL-cholesterol, although less consistently. Blood pressure was unrelated to activity counts or time spent in moderate activity in any of the separate gender-race/ethnicity groups; this finding may be due in part to the fact that treated hypertensives were not excluded. In analyses that did not include BMI these associations were roughly 30-50% larger (data not shown), indicating the substantial confounding of the activity-risk factor association from BMI.

**Table 3:** Partial Correlation Coefficients Between Physical Activity Monitor Measures and Cardiovascular Disease Risk Factors* (P-value) - NHANES 2003-2006, ages 20-65 y (n = 3168)

	Systolic BP	Diastolic BP	Total Cholesterol	HDL-cholesterol	Plasma Glucose**	Hypertension	Diabetes
Activity Counts per min	-0.02	-0.02	0.0076	0.12	-0.07	-0.09	-0.07
	(0.21)	(0.36)	(0.67)	(<.0001)	(0.01)	(<.0001)	(<.0001)
Mod & Vig Activity (min in 1-min bouts)	-0.04	-0.01	-0.00099	0.11	-0.08	-0.07	-0.08
	(0.03)	(0.44)	(0.96)	(<.0001)	(<0.001)	(<.0001)	(<.0001)

*Adjusted for age, sex, race/ethnicity and body mass index

** Plasma glucose concentration adjusted by fasting sampling weights; plasma glucose sample sizes: n = 771 white, n = 306 black, n = 370 Mexican American, n = 1447 total sample

**Table 4:** Change in Risk Factor per Standard Deviation Change in Physical Activity* NHANES 2003-2006, ages 20-65 y (n = 3,168)

Variable	Systolic BP (mmHg)	Diastolic BP (mmHg)	Total Cholesterol (mg/dL)	HDL-cholesterol (mg/dL)	Plasma Glucose** (mg/dL)	Hypertension*** OR	Diabetes*** OR
Mean Counts per min	-0.68	-0.34	0.20	1.56	-1.48	**0.78**	**0.64**
Mod & Vig Activity (min/d in 1-min bouts)	-0.74	-0.07	-0.21	1.49	-1.85	**0.81**	**0.50**

*Adjusted for age, sex, race/ethnicity and body mass index

** Plasma glucose concentration adjusted by fasting sampling weights; plasma glucose sample size: n = 1447 total sample

***Odds ratio per standard deviation change in physical activity

**Table 5:** Partial Correlation Coefficients (P-value) Between Physical Activity Monitor Measures and Cardiovascular Disease Risk Factors* by Gender-Race/Ethnic Groups - NHANES 2003-2006, ages 20-65 y (n = 3168)

	Systolic BP	Diastolic BP	Total Cholesterol	HDL-cholesterol	Plasma Glucose**	Hypertension	Diabetes
**White Males (n = 896)**							
Activity Counts per min	-0.05 (0.17)	-0.04 (0.28)	-0.001 (0.99)	0.18 (<0.001)	-0.06 (0.21)	-0.13 (<0.001)	-0.10 (0.003)
Mod & Vig Activity (min in 1-min bouts)	-0.08 (0.01)	-0.02 (0.50)	-0.002 (0.95)	0.12 (<0.001)	-0.05 (0.34)	-0.11 (0.001)	-0.11 (0.001)
**Black Males (n = 360)**							
Activity Counts per min	-0.02 (0.74)	0.06 (0.22)	0.02 (0.67)	0.05 (0.36)	-0.16 (0.05)	0.09 (0.11)	-0.08 (0.14)
Mod & Vig Activity (min in 1-min bouts)	-0.05 (0.37)	0.05 (0.33)	0.007 (0.89)	0.11 (0.03)	-0.14 (0.08)	0.07 (0.22)	-0.10 (0.05)
**Mexican-American Males (n = 440)**							
Activity Counts per min	0.048 (0.313)	-0.03 (0.59)	-0.002 (0.97)	0.02 (0.66)	0.01 (0.85)	-0.01 (0.81)	-0.01 (0.78)
Mod & Vig Activity (min in 1-min bouts)	0.068 (0.153)	-0.07 (0.14)	-0.02 (0.64)	0.01 (0.81)	-0.05 (0.51)	0.02 (0.74)	-0.02 (0.72)
**White Females (n = 784)**							
Activity Counts per min	-0.03 (0.44)	0.01 (0.76)	0.02 (0.53)	0.11 (0.002)	-0.05 (0.31)	-0.11 (0.003)	-0.04 (0.33)
Mod & Vig Activity (min in 1-min bouts)	-0.04 (0.27)	0.03 (0.44)	-0.02 (0.67)	0.11 (0.002)	-0.09 (0.08)	-0.10 (0.006)	-0.04 (0.22)
**Black Females (n = 329)**							
Activity Counts per min	0.02 (0.71)	0.08 (0.18)	-0.03 (0.61)	0.05 (0.40)	-0.19 (0.02)	-0.02 (0.76)	-0.15 (0.008)
Mod & Vig Activity (min in 1-min bouts)	-0.008 (0.88)	0.02 (0.76)	0.04 (0.45)	0.08 (0.15)	-0.159 (0.05)	-0.01 (0.83)	-0.08 (0.13)
**Mexican-American Females (n = 360)**							
Activity Counts per min	-0.04 (0.44)	-0.08 (0.15)	-0.04 (0.40)	0.04 (0.48)	-0.04 (0.63)	-0.13 (0.01)	-0.06 (0.26)
Mod & Vig Activity (min in 1-min bouts)	-0.05 (0.38)	-0.05 (0.38)	0.01 (0.82)	0.10 (0.05)	-0.10 (0.22)	-0.12 (0.03)	-0.07 (0.17)

*Adjusted for age and BMI

** Plasma glucose concentration adjusted by fasting sampling weights. Plasma glucose sample sizes: n = 771 white, n = 306 black, n = 370 Mexican American, n = 1447 total sample

Activity measures generated by the monitor were also examined for association with relative weight (Table 6); for brevity only moderate activity was included given the virtual absence of vigorous activity. Statistically significant negative associations with BMI were seen in each gender-race/ethnicity group, with the exception of activity counts per minute for black women, and both measures for Mexican-American women. The association with overweight was not consistent, and the nominal relationship was positive and significant in the total sample, albeit weak in magnitude. On the other hand, obesity was negatively associated in all sub-groups for at least one exposure measure, again with the exception of Mexican-American women. The contrasting results in overweight and obesity could in part reflect the fact that mean BMIs were in the overweight range. Likewise, the amount of moderate and vigorous activity in Mexican-American women was the lowest of the sub-groups and the sample size was modest.

**Table 6:** Partial Correlation Coefficients (P-value) - Physical Activity Monitor Measures and BMI, Overweight and Obesity* by Gender-Race/Ethnic Groups - NHANES 2003-2006, ages 20-65 y (n = 3,370)

Variable	Body Mass Index	Overweight	Obesity
**Total Sample (n = 3,370)**			
Activity Counts per min	-0.16 (<0.001)	0.07 (<0.001)	-0.15 (<0.001)
Mod & Vig Activity (min/d in 1-min bouts)	-0.18 (<0.001)	0.10 (<0.001)	-0.18 (<0.001)
**White Males (n = 926)**			
Activity Counts per min	-0.18 (<0.001)	0.05 (0.12)	-0.16 (<0.001)
Mod & Vig Activity (min/d in 1-min bouts)	-0.19 (<0.001)	0.09 (0.006)	-0.20 (<0.001)
**Black Males (n = 386)**			
Activity Counts per min	-0.15 (0.003)	0.05 (0.37)	-0.10 (0.06)
Mod & Vig Activity (min/d in 1-min bouts)	-0.162 (0.001)	0.021 (0.682)	-0.107 (0.036)
**Mexican-American Males (n = 463)**			
Activity Counts per min	-0.21 (<0.001)	0.007 (0.89)	-0.16 (<0.001)
Mod & Vig Activity (min/d in 1-min bouts)	-0.23 (<0.001)	0.004 (0.94)	-0.17 (<0.001)
**White Females (n = 837)**			
Activity Counts per min	-0.19 (<0.001)	0.008 (0.83)	-0.17 (<0.001)
Mod & Vig Activity (min/d in 1-min bouts)	-0.25 (<0.001)	0.02 (0.56)	-0.22 (<0.001)
**Black Females (n = 375)**			
Activity Counts per min	-0.06 (0.27)	0.04 (0.45)	-0.05 (0.39)
Mod & Vig Activity (min/d in 1-min bouts)	-0.14 (0.007)	0.08 (0.11)	-0.15 (0.003)
**Mexican-American Females (n = 383)**			
Activity Counts per min	0.02 (0.72)	0.10 (0.05)	0.03 (0.60)
Mod & Vig Activity (min/d in 1-min bouts)	-0.08 (0.10)	0.06 (0.28)	-0.06 (0.29)

*Adjusted for age

## Discussion

These findings from a large representative sample add a new dimension to our understanding of the patterns and consequences of physical activity in the US population. The estimates of both vigorous and moderate activity were extremely low, and contrast dramatically with those obtained by self-report [8,25-27]. Vigorous activity lasting even 1 minute was only observed in 2% of any of the gender-race/ethnic groups and a 10-minute episode of moderate activity - the intensity obtained by walking up stairs - was recorded in only one third of the participants on any day of monitoring. The overall pattern observed among population sub-groups was, however, consistent with expectations. Mexican-American men were somewhat more active than blacks or whites, which might be attributable to physically demanding occupations, while among women whites appeared to be slightly more active than either blacks or Mexican Americans, possibly reflecting leisure time activity. Activity declined sharply with age; after 60 only ~ 15 minutes of moderate activity was recorded among men and 10 minutes among women per day. Despite the low mean levels a highly significant association was observed between activity level and all the major metabolic risk factors for CVD confirming that the measurements were valid and the effects sufficiently large to confer physiologic consequences.

The major finding from these analyses is the demonstration that population estimates of activity levels from surveys by questionnaire are markedly at variance with those obtained by objective measurements. As the only source available from past surveys, questionnaires have been used in analytic research and have informed public policy for the last 50 years. If the data presented here are correct, a re-evaluation of the conclusions from much of this literature would be required. For example, based on national survey data it was assumed in *Healthy People 2010 *that 23 percent of adults engaged in vigorous activity of more than 20 minutes per episode at least 3 times per week at the beginning of this decade [3]. However, in the NHANES data presented here, < 1 percent of the population achieved this level of expenditure. Likewise, current guidelines recommend 150 minutes of moderate or 75 minutes of vigorous activity per week for adults [6]. Only 0.3%, or 10 of the 3,370 individuals in this sample, achieved that level. This result is in stark contradiction to a recent report using self-reported "usual occupational/domestic activity" in a subset of the same 2003-2006 NHANES participants where 42% of persons with a mean age of 45 met current guidelines [28]; precision of this self-reported activity measure was apparently low, however, since it was unassociated with CVD risk factors. The findings of this recent report are not atypical as current trends based on questionnaires suggest that a large proportion of the population engages in recreational activity; these trends, however, could well be biased by a social desirability effect [6,29,30].

Is the large scale downward shift of the magnitude described here a plausible assessment of activity patterns in the US population? On the surface the discrepancy between questionnaire and measured activity exceeds reasonable expectation. The only measure of validity available from the NHANES survey itself was replication of the risk factor associations. An extensive literature from observational studies and trials supports the association between exercise and CVD risk factors [31-33], therefore replication of these relationships makes it is reasonable to assert that the accelerometer data from NHANES are capturing the physiologic benefit associated with increasing levels of physical activity. Admittedly this validation is indirect, and additional evidence must be sought in external studies which used similar methods. Objective measurement of energy expenditure has only become feasible in the last two decades, and few of the available studies include representative population samples [17,18], therefore we know of no other studies bearing directly on this question. Methodological studies suggest that activity estimates from questionnaires are only correlated at approximately 0.2 with DLW, generally viewed as the most accurate approach [34]. Activity monitors, on the other hand, have been shown to correlate on average at 0.5-0.6 with energy expenditure in activity [14,35,36], representing a substantial increase in precision.

The critical question for these NHANES data, however, is not the degree to which accelerometry places individuals in the correct rank order of increasing activity, but whether the absolute amount of activity is being measured more accurately than by questionnaire. A recent review examined mean differences between estimates from DLW vs. questionnaire in 20 studies [34]. These studies were extremely heterogeneous in terms of sample size, type of participants and the questionnaire and, not surprisingly, the results were highly inconsistent; questionnaires overestimated energy expenditure by 1,000 kcal/day in some instances and under-estimated by 400 kcal/day in others [34]. A similarly heterogeneous literature exists on the concordance between accelerometry and DLW [14]. Contrary to questionnaires, accelerometry slightly under-estimated total expenditure in all but one report, and mean differences tended to be much smaller - in the range of 100 - 200 kcal/day, or about 15 - 25% of physical activity expenditure. A second comprehensive review summarized the concordance between accelerometry and questionnaires in 47 validation studies [37]. On average, questionnaires recorded 44% more daily energy expenditure than did activity monitors. This second review also found that the degree of heterogeneity in the comparison of questionnaires with DLW was so great that no conclusions were possible, although there was an indication that the discrepancy was larger for women than men [37]. It must be recognized that the individual studies reviewed used a variety of instruments, each applying a different algorithm to generate caloric expenditure from activity counts, and they may not be directly comparable to the instrument used in NHANES. In general, however, it seems reasonable to conclude that questionnaires are subject to widely varying bias, most often leading to large over-estimates, while accelerometry has a far smaller, contrary tendency to under-estimation. This evidence would suggest that true expenditure among NHANES participants is closer to the accelerometry estimates, although somewhat higher. Nonetheless, even when applying a threshold of counts per minute that was only 30% of the standard set by direct calorimetry relatively few individuals met the guidelines.

Accelerometry is of course subject to potential biases. For example, cycling or activities that require weight bearing will not be adequately captured, although these are infrequent in the general population. Likewise the device is not worn while swimming. Artifactual increases in counts can also occur as a result of external sources of motion, such as riding in a vehicle. Artifact is a particularly important bias for these data since the vast majority of activity was recorded in episodes lasting only 1 minute. These short bursts are unlikely to represent intentional efforts to accumulate fitness-inducing exercise or physically demanding tasks at work of the type that would be captured by questionnaires. In fact, perhaps the most robust conclusion from this survey is that very few Americans engage in sustained activity, such as jogging or long walks, on a frequent basis. Likewise, in an on-going multi-national survey we have also observed a very low frequency of 10-minute bouts of activity in all 5 study populations (A. Luke, unpublished data). Clearly a more detailed analysis of questionnaire data in concert with accelerometry, and preferably DLW, will be required to resolve these questions.

In addition to the associations with metabolic risk factors, significant but quantitatively weak negative associations exist between the activity measures and BMI and obesity. Likewise, after accounting for BMI, the association with CVD risk factors was substantially weakened, highlighting the confounding that would be expected among these variables. The activity-BMI relationship cannot be considered causal, however, since these data are cross-sectional. Causality could be operating in the opposite direction or in both directions simultaneously. In fact, whether increased activity prevents weight gain is a contentious question. Despite the widely held perception that low levels of energy expenditure in activity is an important risk factor for obesity, prospective data do not support this view [18,38]. Randomized trials, where activity levels are rigorously measured and no attempt is made to restrict calories, likewise show that even substantial increases in energy expenditure in exercise do not result in weight loss because of compensatory increases in intake [39,40]. We conclude, therefore, that the associations observed in the NHANES data presented here between activity and relative weight are spurious - i.e., the direction of the causality is most likely from obesity to lower activity.

Previous analyses of the NHANES activity monitoring data have noted a similar outcome as reported here with regard to levels of activity for the US population and the association with obesity [13,15,41-44]. Troiano et al. [15] and Metzger et al. [13] reported a slightly higher proportion of the US population meeting the current physical activity guidelines than our estimate (i.e., 5%). Metzger et al. used the data to define 5 classes of physical activity, including two classes of very low activity. The combined physical activity level of these 2 classes was less than 25 min of moderate/vigorous physical activity per day and represented almost 79% of the US population [13].

One of the challenges for activity assessment by accelerometry has been the choice of appropriate summary measures. Multiple alternative measures have been used and the results are often difficult to interpret or compare across studies [45-47]. After detailed preliminary analyses we chose three basic metrics - activity counts per minute and time spent in either vigorous or moderate activity. As noted, the 1-minute bouts are subject to over-estimation of activity across the day due to instability of the monitor The 10-minute bouts are potentially meaningful with regard to health benefits, but may not be capturing the true activity patterns of Americans and 2- or 3-minute bouts could yield different results. However, as shown in Figure 2, much less data will be available with these cut-points. Using data from an on-going multi-country study, we estimated an intraclass correlation coefficient of 0.88 with six days of activity monitoring, indicating the NHANES data presented here with an average of 6 days, characterize the individual's activity patterns over the measurement period quite well (A. Luke, unpublished data).

## Conclusion

These analyses of the NHANES physical activity monitor data demonstrate that the US population is sedentary to a degree well beyond what had previously been assumed. Questionnaires on activity, as in most attempts to assess health behaviors, are subject to a strong social desirability bias. Numerous examples exist in the public health literature on behaviors as disparate as smoking and sexual practices [48,49] and it is somewhat surprising in fact that reported data on activity have been taken at face value for so long. Nonetheless the degree of discrepancy exceeds reasonable expectations and unrecognized methodological problems may exist with use of accelerometry in the general population. This detailed analysis of the NHANES results provides a starting point from which to address this important question. Research and surveillance in this vital area will continue to be of limited value until we have access to accurate objective methods of measurement.

## Competing interests

The authors declare that they have no competing interests.

## Authors' contributions

AL, LRD and RSC participated in the design and coordination of the study and helped to draft the manuscript. GC and RD conducted the statistical analyses and helped to draft the manuscript. All authors read and approved the final manuscript.

## Pre-publication history

The pre-publication history for this paper can be accessed here:

http://www.biomedcentral.com/1471-2458/11/387/prepub
